# Supplementary material for: Physical activity is inversely associated with overall cancer risk among college students in the United States: Results from the National College Health Assessment
Source: PLoS One. 2023 Jun 8;18(6):e0287129. doi: 10.1371/journal.pone.0287129 (PMC10249847; doi:10.1371/journal.pone.0287129)
Supplement: Attachment — Submitted filename: Responses to reviewers 05092023.docx [file pone.0287129.s001.docx]

**Authors’ Responses to Editor and Reviewers’ Comments**

**RE: Physical Activity is Inversely Associated with Overall Cancer Risk Among College Students in the United States: Results from the National College Health Assessment**

**Responses to Editor**

1. **Editor**: Please ensure that your manuscript meets PLOS ONE's style requirements, including those for file naming. The PLOS ONE style templates can be found at <https://journals.plos.org/plosone/s/file?id=wjVg/PLOSOne_formatting_sample_main_body.pdf> and 
<https://journals.plos.org/plosone/s/file?id=ba62/PLOSOne_formatting_sample_title_authors_affiliations.pdf>

**Response**: Thank you for your guidance. Please see the revised manuscript which meets PLOS ONE's style requirements.

2. **Editor**: Thank you for submitting the above manuscript to PLOS ONE. During our internal evaluation of the manuscript, we found significant text overlap between your submission and the following previously published works, some of which you are an author.

- <http://pubs.sciepub.com/ajcp/4/1/1/>

We would like to make you aware that copying extracts from previous publications, especially outside the methods section, word-for-word is unacceptable. In addition, the reproduction of text from published reports has implications for the copyright that may apply to the publications.

Therefore, we cannot consider your manuscript as it stands. Please revise the manuscript to rephrase the duplicated text and fully cite all your sources, where appropriate.

We will carefully review your manuscript upon resubmission, so please ensure that your revision is thorough.

Please review your reference list to ensure that it is complete and correct. If you have cited papers that have been retracted, please include the rationale for doing so in the manuscript text, or remove these references and replace them with relevant current references. Any changes to the reference list should be mentioned in the rebuttal letter that accompanies your revised manuscript. If you need to cite a retracted article, indicate the article’s retracted status in the References list and also include a citation and full reference for the retraction notice.

**Response**: Thank you for your comments. The manuscript was revised as suggested, and some duplicated text was rephrased. The reference list was reviewed, and it is complete and correct. There are no cited papers that have been retracted.

**Responses to Reviewer 1**

**Reviewer**: I have carefully read the manuscript and my opinion is that the manuscript has a merit to be published in your reputable journal with some minor corrections. The manuscript is original, informative and readable. The authors aimed to understand the dose-response relation between physical activity and cancer, as well as the associations between meeting US physical activity guidelines and overall cancer risk in US college students. This is the first epidemiological study to examine the association between physical activity and cancer using data from the American College Health Association-National College Health Assessment (ACHA-NCHA) and the feedback might have the great practical implications. The abstract is missing the description of the method precisely. Some of the experienced researchers can reach the relevant information from the method section, but I suggest authors to briefly describe the method of the study as I expect that many young researchers and students will read it, so has to be clearly understandable. The introduction is well written, while materials and methods section is very well prepared and organized according to contemporary methodological rules. At the end, I have no amendments on results and discussion part but I would recommend to the authors to prepare the separate conclusion part in the following order: the main conclusions (with practical implication), the limitations of the study (more precisely) as well as recommendations for the further studies (it is very important to briefly elaborate it and highlight the most important notes). Lastly, I would recommend you to accept this manuscript right after I confirm the authors revise it in the adequate manner.

**Response:** Thank you for your encouraging words and valuable comments. Your suggestions (the method of the study in the abstract and separating conclusion part) have been taken in the revised manuscript (abstract and the first paragraph of the introduction section).

**Responses to Reviewer 2**

**Reviewer**: The manuscript is very well written and the topic is quite interesting for a general audience. The methodology and content are also well done. I recommend that it be accepted with minor revisions. However, I do have some comments.
First, I suggest that in the abstract, you include only the necessary numerical values and limit their use as much as possible.
Secondly, in the introduction, in addition to listing previous studies (3-6) that have examined the given topic, it is important to highlight some relevant findings that are important for the research.
Finally, in the discussion section, I recommend that you relate the results of your study to similar studies and explain the mechanisms behind the obtained results by referring to the existing literature. Expand the discussion and provide a more detailed explanation of the mechanisms underlying the obtained results.

**Response:** Thank you for your encouraging words and valuable comments. The numerical values in the abstract were limited as much as possible as suggested. In the introduction, some relevant findings were highlighted. In the discussion section, although no study examined the association between PA and cancer in U.S. college students, we cited several relevant studies in the second graph and moved the mechanism section from introduction to the discussion section and provided a more detailed explanation of the mechanisms underlying the obtained results as suggested (second paragraph of the discussion section).
